# ERICA: use of screens and consumption of meals and snacks by Brazilian adolescents

**DOI:** 10.1590/S01518-8787.2016050006680

**Published:** 2016-02-02

**Authors:** Juliana Souza Oliveira, Laura Augusta Barufaldi, Gabriela de Azevedo Abreu, Vanessa Sá Leal, Gisela Soares Brunken, Sandra Mary Lima Vasconcelos, Marize Melo dos Santos, Katia Vergetti Bloch

**Affiliations:** I Núcleo de Nutrição. Centro Acadêmico de Vitória. Universidade Federal de Pernambuco. Vitória de Santo Antão, PE, Brasil; II Departamento de Vigilância de Doenças e Agravos Não Transmissíveis e Promoção da Saúde. Secretaria de Vigilância em Saúde.Ministério da Saúde. Brasília, DF, Brasil; III Instituto de Estudos em Saúde Coletiva. Universidade Federal do Rio de Janeiro. Rio de Janeiro, RJ, Brasil; IV Instituto de Saúde Coletiva. Universidade Federal de Mato Grosso. Cuiabá, MT, Brasil; V Faculdade de Nutrição. Universidade Federal de Alagoas. Maceió, AL, Brasil; VI Departamento de Nutrição. Núcleo de Estudos em Saúde Pública. Universidade Federal do Piauí. Teresina, PI, Brasil

**Keywords:** Adolescent, Food Consumption, Attitude to Computers, Television, Cell Phones, Computers, Handheld, Computers, Cross-Sectional Studies

## Abstract

**OBJECTIVE:**

To describe the length of exposure to screens and the prevalence of consumption of meals and snacks by Brazilian adolescents in front of screens.

**METHODS:**

We evaluated 74,589 12 to 17-year old adolescents from 1,247 schools in 124 Brazilian municipalities. A self-administered questionnaire was used. Its segment regarding nutrition contained questions about using TV, computers, and video game systems, having meals while watching TV, and consuming snacks in front of screens. Consumption of meals and snacks in front of screens was analyzed according to the following variables: geographical region, gender, age range, type of school (public or private), and school shift. The prevalences and their respective 95% confidence intervals were estimated under a complex sampling design.

**RESULTS:**

A great deal of the adolescents (73.5%, 95%CI 72.3-74.7) reported spending two or more hours a day in front of screens. That habit was more frequent among male adolescents, private school students, morning shift students, and students from Brazil’s South region. More than half of the adolescents (56.6%, 95%CI 55.4-57.8) reported almost always or always having meals in front of TV, and 39.6% (95%CI 38.8-40.5) of them said they consumed snacks in front of screens exactly as often. Both situations were the most prevalent ones among the girls, who attended public schools and were from Brazil’s Midwest region.

**CONCLUSIONS:**

Length of exposure to screens and consumption of meals and snacks almost always or always in front of screens are high among Brazilian adolescents. It is necessary to develop strategies aiming to reduce the length of screen use, considering the media reality that children and adolescents have been experiencing from earlier and earlier ages. That context must therefore be analyzed in an indissociable way.

## INTRODUCTION

Nutrition habits and preferences originate during early childhood^10^. Throughout the last three decades, globalization has established new paradigms and deep changes in food choices. This scenario, coupled with the increased use of television and other screens, such as video game systems and computers, by children and adolescents^2^ prevents the adoption of healthy lifestyles.

The American Academy of Pediatrics recommends that the time spent in front of TV sets by young people must not exceed 1h to 2h a day^1^; however, that exposure has been observed to be on the rise worldwide. In the United States, in 10 years, the average time spent in front of television by 8 to 18-year-old children and adolescents increased from 3h45 a day to 4h30 a day. Coupled with the hours spent in front of video game systems, computers, and other devices, the time in front of screens increased from 7h30 to almost 11h a day^a^.

The Health Behaviour in School-aged Children (HBSC) Study, which was conducted with European and American adolescents, observed that 61.0% of the 11 to 15-year-old youngsters watched TV for 2h or more a day^26^. In Brazil, *Pesquisa Nacional de Saúde Escolar* (PeNSE – National School Health Survey) showed that 79.5% of ninth-grader students watched TV for two hours or more a day^12^. In turn, *Pesquisa Brasileira de Mídia2015* (Brazilian Media Survey 2015) observed that 16 to 25-year old individuals watch around 4h19 of TV a day^b^.

Among the reasons why screens – including, TV sets, video game systems and computers – have been frequently used are the scarce options for leisure in large urban centers and the children’s parents’ concern regarding their safety, due to the rising violence in cities^14^ ^,^ ^20^. That practice replaced outdoor activities as sources of leisure and entertainment for children and adolescents.

The number of hours in front of TV is directly related to consumption of unhealthy foods throughout the day^10^. As a consequence, the excessive time in front of screens is a risk factor for excess weight gain during early life^2^ ^,^ ^18^.

Among the mechanisms by which the habit of watching TV may affect weight are physical inactivity and increased consumption of obesogenic foods while in front of it^2^ ^,^ ^7^. Additionally, the distraction screens cause interferes in the physiological signs of hunger and satiety, which leads to improper food choices with exaggerated consumption of high-calorie, low-nutrient products^2^.

Another factor contributing to that is the strong influence from the media in food-related behavior. The industry heavily invests on the advertisement of fast foods, high-calorie foods, carbonated beverages, sugary morning cereals, and other highly processed products, which may impact in the formation of food-related habits that increase children and adolescents’ risk of developing chronic diseases^16^.

In that context, this study aimed to describe the length of exposure to screens and the prevalence of consumption of meals and snacks by Brazilian adolescents in front of screens.

## METHODS

The *Estudo de Riscos Cardiovasculares em Adolescentes* (ERICA – Study of Cardiovascular Risks in Adolescents) is a cross-sectional, school-based study, whose objective was to estimate the prevalence of diabetes mellitus, obesity, cardiovascular risk factors, and markers of insulin resistance and inflammation in 12 to 17-year old adolescents attending schools in Brazilian cities with over 100,000 inhabitants. It was conducted between 2013 and 2014.

The 74,589 adolescents from 1,247 schools were analyzed in 124 Brazilian municipalities. The sample was stratified in 32 strata comprising 27 capitals and five sets of municipalities in each of Brazil’s five geographical macro-regions. For each geographical stratum, the larger schools were, the more chances they had of being selected. In turn, the farther away from a state capital a school was, the fewer chances it had of being picked. The sample is representative for medium and large-sized municipalities (> 100 thousand inhabitants) at national and regional levels, and for the Brazilian capitals. The sample design of ERICA is described in further detail in the study by Vasconcellos et al.^25^

All students from the selected groups who agreed to take part in the study and signed consent forms were interviewed and examined. Adolescents outside the age range between 12 and 17 years, pregnant ones, and physically or mentally-challenged adolescents (either temporarily or permanently) were excluded for not being considered eligible. The study was approved by one research ethics committee from each Brazilian state. Details on the experiment protocol are described in Bloch et al.^3^

The information was collected by using self-administered questionnaires in handheld computers. The questionnaire contained around 100 questions divided in 11 blocks: sociodemographic aspects, work, physical activity, diet habits, smoking, use of alcohol, reproductive health, oral health, self-reported morbidity, sleep duration, common mental disorder.

The block about diet habits contained questions on hours using computers, TV, and video game systems on an average week day. Alternatives of answers included: does not perform those activities, performs them for at least 1h a day, performs them from 1h to seven or more hours a day, and does not know or remember. Overuse of screens was defined as watching television, playing video game, or using a computer for more than two hours a day, as recommended by the American Academy of Pediatrics^1^. Studies point out that such behavior is associated with weight gain, low self-esteem, and poor school performance among children and adolescents^2^ ^,^ ^7^ ^,^ ^23^.

This block also contained information regarding having lunch or dinner while watching TV and eating snacks (popcorn, cookies, pretzels, sandwiches, chocolate, or sweets) while watching TV, using a computer, or playing video game. Answer options included no, sometimes, almost every day, and every day. A variable was created to express the act of having meals while watching TV. Thus, variables lunch and dinner in front of TV were combined, in that the following options remained: “never has meals in front of TV”; “sometimes has one of the two meals in front of TV”, and “almost always or always has at least one of the two meals in front of TV”.

Finally, variables “snacks in front of TV” and “snacks in front of computers or video game system” were grouped in “snacks in front of screens”, with three options of answers: “never eats snacks in front of screens”; “sometimes eats snacks in front of screens” and “almost always or always eats snacks in front of screens”.

The unhealthy, food-related habits were analyzed according to the following variables: geographical region (North, Northeast, Midwest, Southeast, and South), gender (male and female), age range (12 to 14 and 15 to 17 years), type of school (public or private), and school shift (morning or afternoon). Analyses were conducted in Stata statistical software, version 14.0, by using the survey mode to analyze the complex-sampling data. Prevalences and their respective 95% confidence intervals (95%CI) were estimated. In the comparison between groups (gender, type of school, school shift, and geographical region), a difference between prevalences was considered to be statistically significant when the 95% confidence intervals (95%CI) of these prevalences did not overlap each other.

## RESULTS

We evaluated 74,589 adolescents, corresponding to 72.9% of the eligible students who were enrolled in the schools and groups selected in the sampling process. The study covered 74.5% of girls and 65.9% of the boys in all studied regions. Separated by region, the least covered one was Midwest (68.3%), and the most covered was South region (81.0%).

Table 1 shows sociodemographic characteristics, distribution through the education network, and school shift of evaluated adolescents. Most of them were females, had an average age of 14.7 years (standard deviation of 1.59), and studied in public schools in the morning shift. In the distribution per geographical regions, the highest and lowest predominance of adolescents were found, respectively, for the Northeast and the South regions.

**Table 1:** Sociodemographic characteristics of the studied population. ERICA, Brazil, 2013-2014.

Variable	n	%
Gender		
Female	41,225	55.3
Male	33,364	44.7
Age (years)		
12-14	34,151	45.8
15-17	40,448	54.2
Type of school		
Public	58,707	78.7
Private	15,882	21.3
School shift		
Morning	53,353	71.5
Afternoon	21,236	28.5
Geographical region		
North	15,073	20.2
Northeast	23,167	31.1
Midwest	9,727	13.0
Southeast	17,080	22.9
South	9,542	12.8

In regards to length of exposure to screens, a great deal of adolescents has reported spending two or more hours a day using TV sets, computers, and video game systems, and that behavior is more prevalent among private school, morning-shift boys living in Southern Brazil. It should be highlighted that such practice was similar among 12 to 14 and 15 to 17-year old adolescents (Table 2).

**Table 2:** Prevalences (%) and 95%CI of exposure to screens for longer than 2h a day in adolescents, according to their sociodemographic characteristics and the ones of their schools. ERICA, Brazil, 2013-2014.

Variable	Overexposure to screens (≥ 2 hours)	

	%	95%CI
Gender		
Female	72.3	70.8-73.7
Male	74.7	73.0-76.4
Age (years)		
12-14	73.2	71.6-74.8
15-17	73.8	72.0-75.6
Type of school		
Public	72.4	71.1-73.6
Private	78.7	75.6-81.5
School shift		
Morning	75.0	73.3-76.6
Afternoon	70.6	68.2-71.9
Geographical region		
North	60.2	58.4-61.9
Northeast	69.2	66.6-71.6
Midwest	73.4	71.8-74.9
Southeast	76.5	74.4-78.6
South	77.9	76.5-79.4

Total	73.5	72.3-74.7

In Table 3, more than half of the adolescents were found to report having meals almost always or always in front of TV, and such habit was the most prevalent among female, public school, morning shift adolescents living in the Midwest region.

**Table 3:** Prevalence (%) of having meals in front of television by adolescents according to their sociodemographic characteristics and the ones of their schools. ERICA, Brazil, 2013-2014.

Variable	Consumption of meals in front of television					

	Never		Sometimes		Almost always or always	

	%	95%CI	%	95%CI	%	95%CI
Gender						
Female	11.8	11.1-12.6	31.0	29.9-32.2	57.1	55.6-58.6
Male	13.8	12.2-15.4	30.2	28.8-31.7	56.0	54.5-57.6
Age (years)						
12-14	12.3	10.9-13.8	30.7	29.7-31.8	57.0	55.3-58.6
15-17	13.4	12.4-14.4	30.5	29.4-31.6	56.1	54.5-57.7
Type of school						
Public	11.3	10.5-12.1	30.5	29.6-31.5	58.2	56.7-59.4
Private	20.0	17.6-22.6	31.0	29.3-32.8	49.0	46.7-51.2
School shift						
Morning	13.2	11.9-14.5	29.8	28.9-30.8	57.0	55.5-58.5
Afternoon	12.0	11.2-13.0	32.4	30.9-33.9	55.6	53.7-57.4
Geographical region						
North	15.5	14.5-16.6	36.5	35.5-37.5	48.0	46.7-49.3
Northeast	11.5	10.1-12.9	34.9	33.3-36.6	53.6	51.3-55.9
Midwest	11.3	10.3-12.5	26.7	25.2-28.3	61.9	60.3-63.5
Southeast	12.0	10.3-13.9	28.4	27.2-29.6	59.7	57.6-61.7
South	17.8	16.3-19.3	30.9	28.5-33.4	51.3	48.3-54.4

Total	12.8	11.8-13.8	30.6	29.8-31.4	56.6	55.4-57.8

Most adolescents reported sometimes eating snacks in front of screens, and approximately 40.0% of them almost always or always do that. This habit was more prevalent among 12 to 14-year-old female private school students living in Midwestern Brazil (Table 4).

**Table 4:** Consumption of snacks in front of television by adolescents according to their sociodemographic characteristics and the ones of their schools. ERICA, Brazil, 2013-2014.

Variable	Consumption of snacks in front of screens					

	Never		Sometimes		Almost always or always	

	%	95%CI	%	95%CI	%	95%CI
Gender						
Female	8.6	8.0-9.4	49.7	48.8-50.6	41.7	40.7-42.6
Male	11.0	10.2-12.0	51.4	50.0-52.7	37.6	36.2-39.0
Age (years)						
12-14	9.2	8.6-9.8	49.5	48.3-50.8	41.3	40.0-42.6
15-17	10.6	9.7-11.6	51.6	50.7-52.6	37.8	36.6-38.9
Type of school						
Public	9.7	9.1-10.4	49.4	48.7-50.2	40.8	40.0-41.7
Private	10.4	9.2-11.6	55.7	54.0-57.4	33.9	32.4-35.4
School shift						
Morning	10.1	9.4-10.9	50.9	50.0-51.8	39.0	38.0-40.0
Afternoon	9.2	8.5-10.1	49.7	48.4-51.2	41.0	39.5-42.6
Geographical region						
North	15.5	14.5-16.5	53.6	52.2-55.0	30.9	29.6-32.3
Northeast	9.6	8.8-10.5	51.1	48.9-53.3	39.3	37.5-41.1
Midwest	8.8	8.1-9.6	47.2	45.3-49.0	44.0	42.1-45.9
Southeast	9.4	8.4-10.5	49.7	48.6-50.8	40.9	39.6-42.3
South	8.7	7.7-9.8	53.2	51.7-54.6	38.1	36.4-39.9

Total	9.8	9.3-10.4	50.5	49.8-51.3	39.6	38.8-40.5

## DISCUSSION

High exposure to screens and the striking custom of having meals and eating snacks in front of them by Brazilian adolescents were observed. Over 70.0% of those youngsters reported spending two hours or more a day in front of TV, computers, or video game systems; approximately 60.0% of them reported having meals almost always or always in front of television; almost 40.0% of them said they ate snacks in front of screens just as often. The distribution of those behaviors was observed to reveal different patterns, according to sociodemographic, school-related, and regional characteristics.

In that sense, ERICA brings an important contribution to mapping out that problem in Brazil, according to its regions. The prevalences of adolescents who almost always watch TV as they have their main meals ranged from 48.0% in the North region to almost 62.0% in the Midwest region. Such habit may be extremely noxious to the general nutrition and health of adolescents, with consequences in adult life.

Studies conducted in the Brazilian territory point towards similar results^4^ ^,^ ^8^ ^,^ ^19^ to the ones identified by ERICA, behaviors which go against the recommendations from the American Academy of Pediatrics^1^. Besides that, studies indicate such exposure in younger age ranges is related to the lowest education level at the age of 26, smoking, low physical aptitude, arterial hypertension, metabolic syndrome, obesity, and high cholesterol in adult life^9^ ^,^ ^13^.

Screens have been occupying central space at the family level, which leads to deep changes in the population’s lifestyle. Traditional habits of families gathered around tables have been replaced by modern habits of having meals in front of screens, which leads people in general to fail to take notice of what they eat and to chew improperly.

In a systematic review^20^, 85.0% of the evaluated articles found a relationship between watching TV and food consumption, and 60.0% of the, between TV and obesity. Longer time watching TV was significantly associated with lower consumption of fruits and vegetables and higher consumption of snacks, sweets, and beverages, and high sugar content^20^. In the study by Momm and Hofelmann^15^, children with abdominal obesity who had meals in front of television (53.6% sometimes and 64.3 always) had worse quality diets.

Time spent watching TV contributes both to sedentary lifestyles and to excess consumption of energy, as children and adolescents who spend hours under that condition are more exposed to advertisement on unhealthy foods. Furthermore, they tend to consume more fast food and soft drinks and less fruits and vegetables than the ones who do not watch as much TV^11^. Using time spent in front of screens as a proxy term for sedentary lifestyles, a study with public school students from Niterói, RJ, Southeastern Brazil, showed that time was significantly associated with excess weight. Their chance of being overweight was 1.864 times higher for students of all ages, and it was higher for boys (OR = 3.195) than for girls (OR = 1.562)^24^.

In ERICA, girls were observed to almost always or always have meals and snacks in front of TV, video game systems, and computers more often as compared to boys. That find may be partly justified through the fact that boys dedicate more time to practicing physical activities, as observed in the study by Vasconcellos et al.^24^, in which they dedicate twice as much time practicing physical activities than girls, considering all ages. On the other hand, in PeNSE, unlike ERICA, girls eat snacks in front of screens less often. Those relations need to be analyzed through models that better clarify the role from those characteristics in food-related behavior^12^.

The analysis of behaviors according to school networks was observed to show high exposure to screens among private school students. That may derive from the fact private school students have more access to technological advances, such as electric and electronic gadgets, and thus they spend more time using those devices^17^. The pattern of prevalences for overuse of screens that was observed in the macro-regions corroborated that result, which was the highest in the most socioeconomically developed regions (Southeast and South).

However, when exposure to screens while having meals and snacks is observed, such habit was more prevalent in public school adolescents. That may be related to their parents’ education levels and work schedules, once family support is important both in fostering their children to have meals on the table and to engage in physical activities and in raising the adolescents’ awareness. Besides that, the lack of public spaces for the practice of physical exercise in large cities is even more concerning in vulnerable social classes, and it contributes to higher rates of people following sedentary lifestyles^6^.

The distribution of those behaviors in the different regions calls attention to the much lower prevalence in the North region, which may be a consequence from the low purchasing power in that region, whereas the low prevalence in the South region may be associated with the education levels of parents. Thus, the association of those habits with different socioeconomic aspects needs to be analyzed by taking into consideration possible confounding variables and effect modifiers, such as income, education levels of parents, and presence of parents in the household and during meals.

The relationship observed in this study, between consumption of snacks in front of screens and the age variable was inversely proportional. A possible explanation is that, as adolescents grow older – mainly the ones from public schools – they spend less time in front of TV, computers, and video game systems, due to their socioeconomic and cultural needs, which lead them to conduct other activities, such as work. However, it is necessary to better understand such relationship. In a meta-analysis study intending on observing the effects from intervention programs targeting to change sedentary lifestyles, Friedrich et al.^5^ suggest that, besides reducing the length of time using TV, computers, and video game systems, focus must be placed on school-based intervention programs. Thus, in that environment, initiatives fostering physical activity and food-related education are required. That is already encompassed by the public policies fostering healthy habits and food safety^c^.

In a systematic review, Schimidt et al.^21^ found that, in most studies, school-based interventions aimed at reducing the time spent in front of screens were observed to have positive results. School, as it is a social environment where teenagers spend most of their time in, is a strategic place for the promotion of educational initiatives and for fostering individuals to adopt healthier lifestyles and keeping them all through adulthood^22^.

Nowadays, two school-based national programs aiming at ensuring the promotion of children and adolescents’ health stand out: National School Food Program (PNAE) and Health at School Program (PSE). Both target students enrolled in public, charity, and community-run schools, and they focus on food safety, promotion of proper food-related habits, and practice of inclusive physical activities.

Another important strategy to fight the early development of risk factors for chronic non-communicable diseases is the effective inclusion of healthy food-related habits in the syllabi of public or private schools, going through all school subjects, and providing for experiences in the daily life of school activities^d^. The same must also be considered regarding physical education classes, in a way that students recognize the importance of practicing physical activities throughout their school years. Moreover, schools need to develop strategies to educate families, emphasizing they are just as responsible for that process, in which their participation is just as important. Hallal et al.^8^ report that the effects from being exposed to sedentary behaviors, such as watching TV, could be minimized in case schools promoted programs which engaged children in out-of-class activities for longer.

Finally, such educational actions cannot dismiss the media reality that children and adolescents have been experiencing from earlier and earlier ages. That context must therefore be analyzed in an indissociable way.

## Footnotes

a Rideout VJ, Foehr UG, Roberts DF. Generation M: media in the lives of 8-to 18- year olds: a Kaiser Family Foundation Study. Menlo Park, CA: The Henry J Kaiser Family Foundation; 2010 [cited 2015 Jul 27]. Available from: http://files.eric.ed.gov/fulltext/ED527859.pdf

b Rideout VJ, Foehr UG, Roberts DF. Generation M: media in the lives of 8-to 18- year olds: a Kaiser Family Foundation Study. Menlo Park, CA: The Henry J Kaiser Family Foundation; 2010 [cited 2015 Jul 27]. Available from: http://files.eric.ed.gov/fulltext/ED527859.pdf

c Ministério do Desenvolvimento Social e Combate à Fome, Secretaria Nacional de Segurança Alimentar e Nutricional. Marco de referência de educação alimentar e nutricional para as políticas públicas. Brasília (DF); 2012 [cited 2015 Jul 25]. Available from: http://www.mds.gov.br/webarquivos/publicacao/seguranca_alimentar/marco_EAN.pdf

d Ministério da Saúde; Ministério da Educação. Portaria interministerial nº 1.010, de 8 de maio de 2006. Institui as diretrizes para a Promoção da Alimentação Saudável nas escolas de educação infantil, fundamental e nível médio das redes públicas e privadas, em âmbito nacional. Brasília (DF); 2006 [cited 2015 Jul 25]. Available from: http://crn3.org.br/legislacao/doc/1.010_2006.pdf

## References

[1] American Academy of Pediatrics, Committee on Public Education (2001). Children, adolescents, and television. Pediatrics.

[2] Bickham DS, Blood EA, Walls CE, Shrier LA, Rich M (2013). Characteristics of screen media use associated with higher BMI in young adolescents. Pediatrics.

[3] Bloch KV, Szklo M, Kuschnir MCC, Abreu GA, Barufaldi LA, Klein CH (2015). The Study of Cardiovascular Risk in Adolescents - ERICA: rationale, design and sample characteristics of a national survey examining cardiovascular risk factor profile in Brazilian adolescents. BMC Public Health.

[4] Dias PJP, Domingos IP, Ferreira MG, Muraro AP, Sichieri R, Gonçalves-Silva RMV (2014). Prevalence and factors associated with sedentary behavior in adolescents. Rev Saude Publica.

[5] Friedrich RR, Polet JP, Schuch I, Wagner MB (2014). Effect of intervention programs in schools to reduce screen time: a meta-analysis. J Pediatr (Rio J).

[6] Frutuoso MFP, Bismarck-Nasr EM, Gambardella AMD (2003). Redução do dispêndio energético e excesso de peso corporal em adolescentes. Rev Nutr.

[7] Ghavamzadeh S, Khalkhali HR, Alizadeh M (2013). TV viewing, independent of physical activity and obesogenic foods, increases overweight and obesity in adolescents. J Health Popul Nutr.

[8] Hallal PC, Knuth AG, Cruz DKA, Mendes MI, Malta DC (2010). Prática de atividade física em adolescentes brasileiros. Cienc Saude Coletiva.

[9] Hancox RJ, Milne BJ, Poulton R (2005). Association of television viewing during childhood with poor educational achievement. Arch Pediatr Adolesc Med.

[10] Hare-Bruun H, Nielsen BM, Kristensen PL, Møller NC, Togo P, Heitmann BL (2011). Television viewing, food preferences, and food habits among children: a prospective epidemiological study. BMC Public Health.

[11] Leal VS, Lira PIC, Menezes RCE, Oliveira JS, Costa EC, Andrade SLLS (2012). Desnutrição e excesso de peso em crianças e adolescentes: uma revisão de estudos brasileiros. Rev Paul Pediatr.

[12] Levy RB, Castro IRR, Cardoso LO, Tavares LF, Sardinha LMV, Gomes FS (2010). Consumo e comportamento alimentar entre adolescentes brasileiros: Pesquisa Nacional de Saúde do Escolar (PeNSE), 2009. Cienc Saude Coletiva.

[13] Mark AE, Janssen I (2008). Relationship between screen time and metabolic syndrome in adolescents. J Public Health (Oxf).

[14] Miranda LL, Souza JA, Santiago MV (2014). A relação lazer e mídia entre adolescentes e jovens de escolas públicas em Fortaleza/CE. Psicol Argum.

[15] Momm N, Hofelmann DA (2014). Qualidade da dieta e fatores associados em crianças matriculadas em uma escola municipal de Itajaí, Santa Catarina. Cad Saude Coletiva.

[16] Moura NC (2010). Influência da mídia no comportamento alimentar de crianças e adolescentes. Segur Alim Nutr.

[17] Oliveira TC, Silva AAM, Santos CJN, Silva JS, Conceição SIO (2010). Atividade física e sedentarismo em escolares da rede pública e privada de ensino em São Luís. Rev Saude Publica.

[18] Rezende LFM, Lopes MR, Rey-López JP, Matsudo VKR, Luiz OC (2014). Sedentary behavior and health outcomes: an overview of systematic reviews. PLoS One.

[19] Ribeiro IC, Taddei JAAC, Colugnatti F (2003). Obesity among children attending elementary public schools in São Paulo, Brazil: a case-control study. Public Health Nutr.

[20] Rossi CE, Albernaz DO, Vasconcelos FAG, Assis MAA, Di Pietro PF (2010). Influência da televisão no consumo alimentar e na obesidade em crianças e adolescentes: uma revisão sistemática. Rev Nutr.

[21] Schmidt ME, Haines J, O’Brien A, McDonald J, Price S, Sherry B (2012). Systematic review of effective strategies for reducing screen time among young children. Obesity.

[22] Telama R, Yang X, Viikari J, Välimäki I, Wanne O, Raitakari O (2005). Physical activity from childhood to adulthood: a 21-year tracking study. Am J Prev Med.

[23] Tremblay MS, LeBlanc AG, Kho ME, Saunders TJ, Larouche R, Colley RC (2011). Systematic review of sedentary behaviour and health indicators in school-aged children and youth. Int J Behav Nutr Phys Act.

[24] Vasconcellos MB, Anjos LA, Vasconcellos MTL (2013). Estado nutricional e tempo de tela de escolares da Rede Pública de Ensino Fundamental de Niterói, Rio de Janeiro, Brasil. Cad Saude Publica.

[25] Vasconcellos MTL, Silva PLN, Szklo M, Kuschnir MCC, Klein CH, Abreu GA (2015). Sampling design for the Study of Cardiovascular Risks in Adolescents (ERICA). Cad Saude Publica.

[26] World Health Organization, Regional Office for Europe (2008). Inequalities in young people’s health: HBSC international report from the 2005/2006 survey.

