# Erratum

**DOI:** 10.1590/S01518-8787.2016050006680err

**Published:** 2016-10-26

In the article “ERICA: use of screens and consumption of meals and snacks by Brazilian adolescents”, DOI:10.1590/S01518-8787.2016050006680, published in the journal “Revista de Saúde Pública”, volume 50 (2016), supplement ERICA, article 7s, in the pages 1, 4 and 6, in items abstract, table 2 and discussion.

Abstract. Page 1. Line 10, where it reads: “(73.5%, 95%CI 72.3-74.7)”, it should read: “(51.8% IC95% 50.7-53.0)”.

Table 2. Page 4. Line 1, column 2, where it reads: “(≥ 2 hours)”, it should read: “(> 2 hours)”.

Table 2. Page 4. It should read the values presented below:

**Table:** 

Characteristics	Overexposure to screens (> 2 hours)	

	%	95%CI
Gender		
Female	50.9	49.8-52.1
Male	52.7	50.9-54.4
Age (years)		
12 to 14	50.5	49.1-51.8
15 to 17	53.3	51.6-54.9
Type of school		
Public	50.9	49.6-52.1
Private	56.3	54.9-57.7
School shift		
Morning	54.0	52.5-55.4
Afternoon	47.1	45.2-49.0
Geographical region		
North	39.3	37.7-40.9
Northeast	46.6	43.8-49.4
Central West	52.4	50.8-53.9
Southeast	54.3	52.5-56.2
South	59.1	57.4-60.7

Total	51.8	50.7-53.0

Discussion. Page 6. Line 2, where it reads: “Over 70.0%, it should read: “Over 50.0%”.

