# Supplementary material for: ERICA: use of screens and consumption of meals and snacks by Brazilian adolescents
Source: Rev Saude Publica. 2016 Feb 2;50(Suppl 1):7s. doi: 10.1590/S01518-8787.2016050006680 (PMC4767035; doi:10.1590/S01518-8787.2016050006680)
Supplement: Translation

# Translation: ERICA: uso de telas e consumo de refeições e petiscos por adolescentes brasileiros

**Authors:** Juliana Souza Oliveira, Laura Augusta Barufaldi, Gabriela de Azevedo Abreu, Vanessa Sá Leal, Gisela Soares Brunken, Sandra Mary Lima Vasconcelos, Marize Melo dos Santos, Katia Vergetti Bloch

**Affiliations:** I Núcleo de Nutrição. Centro Acadêmico de Vitória. Universidade Federal de Pernambuco. Vitória de Santo Antão, PE, Brasil; II Departamento de Vigilância de Doenças e Agravos Não Transmissíveis e Promoção da Saúde. Secretaria de Vigilância em Saúde. Ministério da Saúde. Brasília, DF, Brasil; III Instituto de Estudos em Saúde Coletiva. Universidade Federal do Rio de Janeiro. Rio de Janeiro, RJ, Brasil; IV Instituto de Saúde Coletiva. Universidade Federal de Mato Grosso. Cuiabá, MT, Brasil; V Faculdade de Nutrição. Universidade Federal de Alagoas. Maceió, AL, Brasil; VI Departamento de Nutrição. Núcleo de Estudos em Saúde Pública. Universidade Federal do Piauí. Teresina, PI, Brasil

**Keywords:** Adolescente, Consumo de Alimentos, Atitude Frente aos Computadores, Televisão, Telefones Celulares, Computadores de Mão, Computadores, Estudo Transversal

## Abstract

**OBJETIVO:**

Descrever o tempo de exposição às telas e a prevalência de consumo de refeições e petiscos em frente a telas em adolescentes brasileiros.

**MÉTODOS:**

Foram avaliados 74.589 adolescentes de 12 a 17 anos de 1.247 escolas em 124 municípios brasileiros. Foi utilizado questionário autopreenchível, cujo bloco sobre alimentação continha perguntas sobre o uso de TV, computador e videogames, realização de refeições assistindo TV e consumo de petiscos em frente a telas. O consumo de refeições e petiscos em frente a telas foi analisado segundo as variáveis: região geográfica, sexo, faixa etária, tipo de escola (pública ou particular) e turno de estudo. Foram estimadas prevalências e respectivos intervalos de confiança de 95%, considerando-se o desenho de amostra complexa.

**RESULTADOS:**

Grande parte dos adolescentes (73,5%, IC95% 72,3-74,7) referiu passar duas ou mais horas por dia em frente às telas. Esse hábito foi mais frequente entre os adolescentes do sexo masculino, de escola particular, do turno da manhã e da região Sul do País. Mais da metade dos adolescentes (56,6%, IC95% 55,4-57,8) relatou realizar as refeições quase sempre ou sempre em frente à TV, e 39,6% (IC95% 38,8-40,5) consumiam petiscos em frente às telas com essa mesma frequência. Ambas as situações foram mais prevalentes entre as meninas, que estudavam na rede pública de ensino e da região Centro-Oeste.

**CONCLUSÕES:**

O tempo de exposição às telas e o consumo de refeições e petiscos quase sempre ou sempre em frente a telas é elevado entre os adolescentes brasileiros. Faz-se necessário desenvolver estratégias que visem à redução do tempo de uso de telas, considerando a realidade midiática que crianças e adolescentes têm vivenciado de forma cada vez mais precoce, devendo-se, portanto, trabalhar esse contexto de forma indissociável.

## INTRODUÇÃO

Hábitos e preferências alimentares são originados na primeira infância^10^. Ao longo das três últimas décadas, a globalização estabeleceu novos paradigmas e profundas alterações nas escolhas alimentares. Esse cenário associado ao aumento no uso da televisão e outras telas, como videogames e computadores, por crianças e adolescentes^2^ compromete a adoção de um estilo de vida saudável.

A Academia Americana de Pediatria preconiza que o tempo dispendido em frente à TV pelos jovens não deva ultrapassar 1h a 2h por dia^1^; no entanto, observa-se aumento crescente desta exposição em nível mundial. Nos Estados Unidos, em 10 anos, o tempo médio gasto em frente à televisão por crianças e adolescentes de oito a 18 anos de idade aumentou de 3h45min para 4h30min por dia, e, somado às horas despendidas com videogame, computador, e outras mídias, o tempo em frente a telas aumentou de 7h30min para quase 11h diárias^a^.

A Pesquisa *Health Behaviour in School-aged Children* (HBSC), realizada com adolescentes europeus e da América do Norte, observou que 61,0% a 68,0% dos jovens entre 11 e 15 anos assistiam 2h ou mais de TV por dia^26^. No Brasil, a Pesquisa Nacional de Saúde Escolar (PeNSE) mostrou que 79,5% dos escolares do nono ano no ensino fundamental assistiam TV por duas ou mais horas diárias^12^. Já a Pesquisa Brasileira de Mídia 2015 observou que os indivíduos entre 16 e 25 anos assistem cerca de 4h19min de TV por dia^b^.

Dentre os motivos pelos quais as telas, incluindo TV, videogames e computadores, vêm sendo utilizadas com frequência estão as poucas opções de lazer nos grandes centros urbanos e a preocupação dos pais com a segurança de seus filhos, devido à crescente violência nas cidades^14^ ^,^ ^20^. Essa prática substituiu as atividades externas como fonte de lazer e entretenimento de crianças e adolescentes.

O número de horas diante da TV está diretamente relacionado ao consumo de alimentos não saudáveis ao longo do dia^10^. Como consequência, o tempo excessivo em frente às telas é fator de risco para o desenvolvimento de excesso de peso em fases precoces da vida^2^ ^,^ ^18^.

Dentre os mecanismos pelos quais o hábito de assistir TV pode afetar o peso estão a inatividade física e o maior consumo de alimentos obesogênicos durante o tempo gasto diante da televisão^2^ ^,^ ^7^. Adicionalmente, a distração provocada pelas telas interfere nos sinais fisiológicos de fome e saciedade, levando a escolhas alimentares inadequadas com consumo exacerbado de produtos de elevado teor calórico e baixo teor de nutrientes^2^.

Outro fator contribuinte é a forte influência da mídia no comportamento alimentar. As indústrias investem fortemente em propagandas de *fast-food*, alimentos ricos em calorias, bebidas carbonatadas, cereais matinais açucarados e outros produtos ultraprocessados, o que pode impactar na formação de hábitos alimentares de risco para o desenvolvimento de doenças crônicas em crianças e adolescentes^16^.

Nesse contexto, o objetivo deste estudo foi descrever o tempo de exposição às telas e a prevalência de consumo de refeições e petiscos em frente às telas em adolescentes brasileiros.

## MÉTODOS

O Estudo de Riscos Cardiovasculares em Adolescentes (ERICA) é um estudo transversal, de âmbito nacional de base escolar, cujo objetivo foi estimar a prevalência do diabetes mellitus, obesidade, fatores de risco cardiovascular e de marcadores de resistência à insulina e inflamatórios em adolescentes de 12 a 17 anos que frequentam escolas em cidades brasileiras com mais de 100.000 habitantes, conduzido nos anos de 2013 e 2014.

Foram avaliados 74.589 adolescentes de 1.247 escolas em 124 municípios brasileiros. A amostra foi estratificada em 32 estratos constituídos por 27 capitais e cinco conjuntos de municípios em cada uma das cinco macrorregiões geográficas do País. Para cada estrato geográfico, as escolas foram selecionadas com probabilidade proporcional ao tamanho e inversamente proporcional à distância da capital. A amostra é representativa para municípios de médio e grande porte (> 100 mil habitantes) em âmbito nacional, regional e para as capitais brasileiras. No estudo de Vasconcellos et al.^25^ o desenho da amostra do ERICA é descrito com mais detalhes.

Foram entrevistados e examinados todos os alunos das turmas selecionadas que concordaram em participar do estudo e assinaram o termo de assentimento. Foram excluídos das análises, por não serem considerados elegíveis, os adolescentes que não pertenciam à faixa etária de 12 a 17 anos, adolescentes grávidas e aqueles com deficiência física ou mental, temporária ou permanente. O estudo foi aprovado por um comitê de ética em pesquisa de cada estado brasileiro. Detalhes sobre o protocolo do estudo são descritos em Bloch et al.^3^

As informações foram coletadas usando-se questionário autopreenchível no coletor eletrônico de dados *personal digital assistant* (PDA). O questionário continha cerca de 100 questões divididas em 11 blocos: aspectos sociodemográficos, trabalho, atividade física, alimentação, tabagismo, uso de bebidas alcoólicas, saúde reprodutiva, saúde bucal, morbidade referida, duração do sono, transtorno mental comum.

O bloco sobre alimentação continha perguntas sobre as horas de uso de computador, TV e videogame em um dia de semana comum. As alternativas de respostas incluíam: não realiza essas atividades, realiza por menos de 1h por dia, de 1h até sete ou mais horas por dia e não sabia ou lembrava. O tempo excessivo de tela foi definido como assistir televisão, jogar videogame ou usar o computador por mais de duas horas por dia, conforme recomenda a Academia Americana de Pediatria^1^. Estudos apontam que esse comportamento está associado ao aumento da composição corporal, à baixa autoestima e à redução no desempenho escolar entre crianças e adolescentes^2^ ^,^ ^7^ ^,^ ^23^.

Este bloco englobava ainda informações sobre almoçar ou jantar assistindo TV e sobre comer petiscos (pipoca, biscoitos, salgadinhos, sanduíches, chocolates ou balas) assistindo TV, usando computador ou jogando videogame. As opções de respostas foram: não, às vezes, quase todos os dias e todos os dias. Para a análise, foi criada uma variável para expressar a realização de refeições assistindo TV. Assim, combinaram-se as variáveis almoço e jantar em frente à TV, de forma que permaneceram as opções: “nunca faz refeições em frente à TV”; “às vezes faz uma ou duas das refeições em frente à TV” e “faz quase sempre ou sempre pelo menos uma das refeições em frente à TV”.

Por fim, as variáveis, “petisco em frente à TV” e “petisco em frente ao computador ou videogame” foram agrupadas em “petisco em frente a telas”, com três opções de resposta: “nunca come petisco em frente a telas”; “às vezes come petisco em frente a telas” e “quase sempre ou sempre come petisco em frente a telas”.

Os comportamentos não saudáveis relacionados à alimentação foram analisados segundo as seguintes variáveis: região geográfica (Norte, Nordeste, Centro-Oeste, Sudeste e Sul), sexo (masculino e feminino), faixa etária (12-14 e 15-17 anos), tipo de escola (pública ou particular) e turno de estudo (manhã ou tarde). As análises foram realizadas no programa estatístico Stata, versão 14.0, utilizando-se o módulo *survey* para análise de dados de amostra complexa. Foram estimadas prevalências e respectivos intervalos de confiança de 95% (IC95%). Na comparação entre grupos (sexo, grupo etário, tipo de escola, turno de estudo e região geográfica), foi considerada diferença estatisticamente significativa entre prevalências quando os intervalos de confiança de 95% (IC95%) destas não se sobrepuseram.

## RESULTADOS

Foram avaliados 74.589 adolescentes, que correspondem a 72,9% dos estudantes elegíveis matriculados nas escolas e turmas selecionadas no processo de amostragem. A cobertura por sexo foi de 74,5% nas meninas e 65,9% nos meninos e, por região, a menor foi no Centro-Oeste (68,3%) e a maior, no Sul (81,0%).

A Tabela 1 apresenta as características sociodemográficas, distribuição pela rede de ensino e turno de estudo dos adolescentes avaliados. A maior parte era do sexo feminino, com média de idade de 14,7 anos (desvio padrão de 1,59), estudava em escolas públicas e no turno da manhã. Na distribuição por região geográfica, observou-se maior e menor predomínio de adolescentes do Nordeste e Sul, respectivamente.

**Tabela 1:** Características sociodemográficas da população de estudo. ERICA, Brasil, 2013-2014.

Variável	n	%
Sexo		
Feminino	41.225	55,3
Masculino	33.364	44,7
Idade (anos)		
12-14	34.151	45,8
15-17	40.448	54,2
Tipo de escola		
Pública	58.707	78,7
Particular	15.882	21,3
Turno de estudo		
Manhã	53.353	71,5
Tarde	21.236	28,5
Região geográfica		
Norte	15.073	20,2
Nordeste	23.167	31,1
Centro-Oeste	9.727	13,0
Sudeste	17.080	22,9
Sul	9.542	12,8

No que diz respeito ao tempo de tela, a grande parte dos adolescentes referiu passar duas ou mais horas do dia fazendo uso de TV, computador e videogame, sendo esse comportamento mais prevalente entre os meninos, de escolas particulares, do turno da manhã e que residiam na região Sul do País. Destaca-se que essa prática foi similar entre adolescentes de 12 a 14 e 15 a 17 anos de idade (Tabela 2).

**Tabela 2:** Prevalências (%) e IC95% de tempo de telas maior que 2h/dia em adolescentes segundo características sociodemográficas e da escola. ERICA, Brasil, 2013-2014.

Variável	Exposição excessiva em frente a telas (≥ 2 horas)	

	%	IC95%
Sexo		
Feminino	72,3	70,8-73,7
Masculino	74,7	73,0-76,4
Idade (anos)		
12-14	73,2	71,6-74,8
15-17	73,8	72,0-75,6
Tipo de escola		
Pública	72,4	71,1-73,6
Particular	78,7	75,6-81,5
Turno de estudo		
Manhã	75,0	73,3-76,6
Tarde	70,6	68,2-71,9
Região geográfica		
Norte	60,2	58,4-61,9
Nordeste	69,2	66,6-71,6
Centro-Oeste	73,4	71,8-74,9
Sudeste	76,5	74,4-78,6
Sul	77,9	76,5-79,4

Total	73,5	72,3-74,7

Na Tabela 3, verifica-se que mais da metade dos adolescentes relatou realizar as refeições quase sempre ou sempre em frente à TV, sendo essa prática mais prevalente entre os adolescentes do sexo feminino, que estudavam na rede pública de ensino, no turno da manhã e pertencentes à região Centro-Oeste.

**Tabela 3:** Prevalência (%) de realização de refeições em frente à televisão por adolescentes segundo características sociodemográficas e da escola. ERICA, Brasil, 2013-2014.

Variável	Consumo de refeições em frente à televisão					

	Nunca		Às vezes		Quase sempre ou sempre	
		
	%	IC95%	%	IC95%	%	IC95%
Sexo						
Feminino	11,8	11,1-12,6	31,0	29,9-32,2	57,1	55,6-58,6
Masculino	13,8	12,2-15,4	30,2	28,8-31,7	56,0	54,5-57,6
Idade (anos)						
12-14	12,3	10,9-13,8	30,7	29,7-31,8	57,0	55,3-58,6
15-17	13,4	12,4-14,4	30,5	29,4-31,6	56,1	54,5-57,7
Tipo de escola						
Pública	11,3	10,5-12,1	30,5	29,6-31,5	58,2	56,7-59,4
Particular	20,0	17,6-22,6	31,0	29,3-32,8	49,0	46,7-51,2
Turno de estudo						
Manhã	13,2	11,9-14,5	29,8	28,9-30,8	57,0	55,5-58,5
Tarde	12,0	11,2-13,0	32,4	30,9-33,9	55,6	53,7-57,4
Região geográfica						
Norte	15,5	14,5-16,6	36,5	35,5-37,5	48,0	46,7-49,3
Nordeste	11,5	10,1-12,9	34,9	33,3-36,6	53,6	51,3-55,9
Centro-Oeste	11,3	10,3-12,5	26,7	25,2-28,3	61,9	60,3-63,5
Sudeste	12,0	10,3-13,9	28,4	27,2-29,6	59,7	57,6-61,7
Sul	17,8	16,3-19,3	30,9	28,5-33,4	51,3	48,3-54,4

Total	12,8	11,8-13,8	30,6	29,8-31,4	56,6	55,4-57,8

A maior parte dos adolescentes referiu consumir petiscos em frente a telas às vezes, e aproximadamente 40,0% quase sempre ou sempre adotam tal prática. Este hábito foi mais prevalente entre as meninas, com idade entre 12 e 14 anos, estudantes de escolas públicas e residentes na região Centro-Oeste do País (Tabela 4).

**Tabela 4:** Consumo de petiscos em frente a telas por adolescentes, segundo características sociodemográficas e da escola. ERICA, Brasil, 2013-2014.

Variável	Consumo de petiscos em frente às telas					

	Nunca		Às vezes		Quase sempre ou sempre	
		
	%	IC95%	%	IC95%	%	IC95%
Sexo						
Feminino	8,6	8,0-9,4	49,7	48,8-50,6	41,7	40,7-42,6
Masculino	11,0	10,2-12,0	51,4	50,0-52,7	37,6	36,2-39,0
Idade (anos)						
12-14	9,2	8,6-9,8	49,5	48,3-50,8	41,3	40,0-42,6
15-17	10,6	9,7-11,6	51,6	50,7-52,6	37,8	36,6-38,9
Tipo de escola						
Pública	9,7	9,1-10,4	49,4	48,7-50,2	40,8	40,0-41,7
Particular	10,4	9,2-11,6	55,7	54,0-57,4	33,9	32,4-35,4
Turno de estudo						
Manhã	10,1	9,4-10,9	50,9	50,0-51,8	39,0	38,0-40,0
Tarde	9,2	8,5-10,1	49,7	48,4-51,2	41,0	39,5-42,6
Região geográfica						
Norte	15,5	14,5-16,5	53,6	52,2-55,0	30,9	29,6-32,3
Nordeste	9,6	8,8-10,5	51,1	48,9-53,3	39,3	37,5-41,1
Centro-Oeste	8,8	8,1-9,6	47,2	45,3-49,0	44,0	42,1-45,9
Sudeste	9,4	8,4-10,5	49,7	48,6-50,8	40,9	39,6-42,3
Sul	8,7	7,7-9,8	53,2	51,7-54,6	38,1	36,4-39,9

Total	9,8	9,3-10,4	50,5	49,8-51,3	39,6	38,8-40,5

## DISCUSSÃO

Observou-se elevada exposição às telas e o costume marcante da realização de refeições e consumo de petiscos em frente às mesmas por adolescentes brasileiros. Mais de 70,0% desses jovens relataram passar duas ou mais horas por dia em frente à TV, computador ou videogames; aproximadamente 60,0% referiram realizar as refeições quase sempre ou sempre em frente à televisão; quase 40,0% disseram consumir petiscos em frente às telas com a mesma regularidade. A distribuição desses comportamentos revelou padrões diferentes, de acordo com as características sociodemográficas, da escola e regionais.

Nesse sentido, o ERICA traz importante contribuição para o mapeamento do problema no Brasil, segundo as regiões. As prevalências de adolescentes que quase sempre assistem TV enquanto realizam as principais refeições variou de 48,0% na região Norte a quase 62,0% na região Centro-Oeste. Esse hábito pode ser extremamente prejudicial à nutrição e saúde em geral dos adolescentes, com repercussões na fase adulta.

Pesquisas realizadas no território brasileiro apontam resultados semelhantes^4^ ^,^ ^8^ ^,^ ^19^ aos identificados no ERICA, comportamentos que estão em desacordo ao preconizado pela Academia Americana de Pediatria^1^. Além disso, estudos indicam que essa exposição em grupos etários mais jovens se relaciona ao menor nível de escolaridade aos 26 anos, tabagismo, baixa aptidão física, hipertensão arterial, síndrome metabólica, obesidade e colesterol elevado na fase adulta^9^ ^,^ ^13^.

As telas têm ocupado lugar de destaque no âmbito familiar, levando a profundas mudanças no estilo de vida da população. Hábitos tradicionais de reuniões familiares ao redor da mesa vêm sendo substituídos por hábitos modernos de alimentar-se em frente a telas, levando as pessoas, em geral, a não prestarem atenção no que consomem e não mastigarem de maneira adequada.

Em revisão sistemática^20^, 85,0% dos artigos avaliados encontraram relação entre TV e consumo de alimentos e 60,0% dos artigos, entre TV e obesidade. Maior tempo assistindo TV foi significativamente associado ao menor consumo de frutas e verduras e maior consumo de porções de salgadinhos, doces e bebidas e elevado teor de açúcar^20^. No estudo de Momm e Hofelmann^15^, as crianças com obesidade abdominal e que realizavam refeições em frente à televisão (53,6% às vezes e 64,3% sempre) tiveram pior qualidade da dieta.

O tempo assistindo à TV contribui tanto para o sedentarismo como para o consumo excessivo de energia, uma vez que, além de passar horas sob essa situação, crianças e adolescentes estão mais expostas à propaganda de alimentos não saudáveis. Ademais, tendem a consumir mais *fast-food* e refrigerantes e menos frutas e vegetais do que aquelas que passam menos tempo dedicado à TV^11^. Utilizando o tempo em frente à tela como *proxy* de sedentarismo, um estudo com escolares da rede pública de Niterói, RJ, mostrou que esse tempo associou-se significativamente ao excesso de peso. A chance de apresentar excesso de peso foi 1,864 vezes maior para os escolares de todas as idades, sendo maior para os meninos (OR = 3,195) do que para as meninas (OR = 1,562)^24^.

No ERICA, as meninas consumiram com maior frequência sempre ou quase sempre refeições e petiscos em frente à TV, videogames e computadores, quando comparadas aos meninos. Esse achado pode ser justificado, em parte, pelo fato de meninos destinarem mais tempo à prática de atividade física, como observado no estudo de Vasconcellos et al.^24^ em que eles dedicavam o dobro de tempo realizando atividade física em relação às meninas, considerando todas as idades. Por outro lado, na PeNSE, ao contrário do ERICA, as meninas consomem em menor frequência petiscos frente a telas. Essas relações precisam ser analisadas com modelos que esclareçam melhor o papel dessas características no comportamento alimentar^12^.

A análise dos comportamentos em relação à rede de ensino indicou elevada exposição às telas entre escolares de escolas particulares. Isso pode ser consequência de os estudantes de escolas privadas terem mais acesso aos avanços tecnológicos, como os elétricos e eletrônicos e, assim, ficarem mais tempo em uso desses equipamentos^17^. O padrão de prevalências de tempo excessivo de telas observado nas macrorregiões foi coerente com esse resultado, maior nas regiões de maior desenvolvimento socioeconômico (Sudeste e Sul).

Entretanto, quando observada a exposição às telas durante o consumo de refeições e petiscos, esse hábito foi maior em adolescentes de escolas públicas. Isso pode estar relacionado ao nível de instrução e à rotina de trabalho dos pais, uma vez que, para a realização das refeições à mesa e para o incentivo à prática de atividade física, o apoio familiar é importante, tanto quanto a conscientização dos próprios adolescentes. Além disso, a carência de espaços públicos nas grandes cidades para a realização de exercícios físicos é ainda maior nas classes sociais vulneráveis e contribui para taxas mais elevadas de comportamentos sedentários^6^.

A distribuição desses comportamentos nas diferentes regiões chama a atenção à prevalência bem menor na região Norte, que pode ser reflexo de baixo poder aquisitivo nessa região, ao passo que a baixa prevalência na região Sul pode estar associada à escolaridade dos pais. Assim, a associação desses hábitos com diferentes aspectos socioeconômicos precisa ser analisada levando em consideração possíveis variáveis de confundimento e modificadoras de efeito, tais como renda, escolaridade dos pais e presença dos pais na residência e durante as refeições.

A relação observada neste estudo entre consumo de petiscos em frente a telas e a variável idade foi inversa. Uma possível explicação é que, com o aumento da idade, o adolescente, sobretudo aqueles de escolas públicas, fica menos tempo em frente à TV, computador e videogames, devido às exigências socioeconômicas e culturais, que os levam a exercer outras atividades, como o trabalho. No entanto, é necessário compreender melhor essa relação. Em estudo de meta-análise visando verificar os efeitos dos programas de intervenção na mudança do comportamento sedentário, Friedrich et al.^5^ sugerem que, além de reduzir o tempo de uso de TV, computadores e videogames, deve-se focar em programas de intervenção no âmbito escolar. Assim, nesse ambiente, é necessário estímulo e promoção da atividade física e em ações de educação alimentar e nutricional, a qual está entre as políticas públicas de promoção da saúde e da segurança alimentar e nutricional^c^.

Em revisão sistemática, Schimidt et al.^21^ constataram que, na maioria dos estudos, intervenções no âmbito escolar para diminuir o tempo em frente à tela mostraram resultados positivos. A escola, por ser um ambiente social onde os adolescentes passam boa parte de seu tempo, constitui um espaço estratégico para promover práticas educativas e incentivar os indivíduos a adotarem hábitos de vida mais saudáveis e mantê-los durante a vida adulta^22^.

Atualmente, destacam-se dois programas nacionais de âmbito escolar que objetivam garantir a promoção da saúde de crianças e adolescentes: o Programa Nacional de Alimentação Escolar (PNAE) e o Programa Saúde na Escola (PSE). Ambos atendem estudantes matriculados em escolas públicas, filantrópicas e em entidades comunitárias e estão voltados à segurança alimentar, promoção da alimentação adequada e saudável e prática de atividade física inclusiva.

Outra estratégia importante de combate precoce aos fatores de risco de doenças crônicas não transmissíveis é a inserção efetiva do tema alimentação saudável no projeto político pedagógico das escolas, sejam públicas ou privadas, perpassando todas as áreas de estudo e propiciando experiências no cotidiano das atividades escolares^d^. Ele também deve ser considerado em relação às aulas de educação física, de modo que os escolares reconheçam a importância da prática de atividade física de forma contínua no currículo escolar. Ademais, as escolas precisam desenvolver estratégias de informação às famílias, enfatizando sua corresponsabilidade e a importância de sua participação nesse processo. Hallal et al.^8^ relatam que os efeitos da exposição a comportamentos sedentários, como assistir TV, poderiam ser minimizados caso as escolas promovessem programas que utilizassem maior tempo das crianças em atividades extraclasse.

Por fim, as ações educativas não devem desconsiderar a realidade midiática que crianças e adolescentes têm vivenciado de forma cada vez mais precoce, devendo-se, portanto, trabalhar esse contexto de forma indissociável.

## Footnotes

a Rideout VJ, Foehr UG, Roberts DF. Generation M: media in the lives of 8-to 18-year olds: a Kaiser Family Foundation Study. Menlo Park, CA: The Henry J Kaiser Family Foundation; 2010 [citado 2015 jul 27]. Disponível em: http://files.eric.ed.gov/fulltext/ED527859.pdf

b Presidência da República (BR), Secretaria de Comunicação Social. Pesquisa Brasileira de Mídia 2015: hábitos de consumo de mídia pela população brasileira. Brasília (DF): Secom; 2014 [citado 2015 jul 27]. Disponível em: http://www.secom.gov.br/atuacao/pesquisa/lista-de-pesquisas-quantitativas-e-qualitativas-de-contratos-atuais/pesquisa-brasileira-de-midia-pbm-2015.pdf

c Ministério do Desenvolvimento Social e Combate à Fome (BR), Secretaria Nacional de Segurança Alimentar e Nutricional. Marco de referência de educação alimentar e nutricional para as políticas públicas. Brasília (DF); 2012 [citado 2015 jul 25]. Disponível em: http://www.mds.gov.br/webarquivos/publicacao/seguranca_alimentar/marco_EAN.pdf

d Ministério da Saúde; Ministério da Educação. Portaria interministerial nº 1.010, de 8 de maio de 2006. Institui as diretrizes para a Promoção da Alimentação Saudável nas escolas de educação infantil, fundamental e nível médio das redes públicas e privadas, em âmbito nacional. Brasília (DF); 2006 [citado 2015 jul 25]. Disponível em: http://crn3.org.br/legislacao/doc/1.010_2006.pdf
